# Comparison of two lab-scale protocols for enhanced mRNA-based CAR-T cell generation and functionality

**DOI:** 10.1038/s41598-023-45197-x

**Published:** 2023-10-24

**Authors:** Nadine von Auw, Robert Serfling, Reni Kitte, Nadja Hilger, Chengkang Zhang, Clara Gebhardt, Anna Duenkel, Paul Franz, Ulrike Koehl, Stephan Fricke, U. Sandy Tretbar

**Affiliations:** 1 Department for Cell and Gene Therapy Development, Fraunhofer Institute for Cell Therapy and Immunology (IZI), Perlickstr. 1, 04103 Leipzig, Germany; 2 Lonza, 9900 Medical Center Drive, Rockville, MD 20850 USA; 3 Fraunhofer Cluster of Excellence Immune-Mediated Diseases CIMD, Leipzig, Germany; 4 Fraunhofer Institute for Cell Therapy and Immunology (IZI), Perlickstr. 1, 04103 Leipzig, Germany; 5 Medical Faculty, Institute for Clinical Immunology, University of Leipzig, Johannisallee 30, 04103 Leipzig, Germany

**Keywords:** Cancer, Immunology, Molecular biology, Molecular medicine

## Abstract

Process development for transferring lab-scale research workflows to automated manufacturing procedures is critical for chimeric antigen receptor (CAR)-T cell therapies. Therefore, the key factor for cell viability, expansion, modification, and functionality is the optimal combination of medium and T cell activator as well as their regulatory compliance for later manufacturing under Good Manufacturing Practice (GMP). In this study, we compared two protocols for CAR-mRNA-modified T cell generation using our current lab-scale process, analyzed all mentioned parameters, and evaluated the protocols’ potential for upscaling and process development of mRNA-based CAR-T cell therapies.

## Introduction

Chimeric antigen receptor (CAR)-modified T cells are therapeutically used in the treatment of hematological malignancies, with six approved CAR-T cell products in 2023^1^. In general, the CAR-T cell manufacturing process is an ex vivo culture and modification process that includes leukapheresis of patient-specific T cells, T cell activation, genetic modification by viral transduction, CAR-T cell expansion, cryopreservation, and reinfusion of the final product into the patient^1,2^. An alternative to viral transduction is the delivery of CAR-encoding mRNA as an in vitro transcript (IVT) for non-integrating, transient modification of T cells^3^, which has already been tested in several clinical trials^4–10^. For ex vivo generation of mRNA-based CAR-T cells, a slightly different manufacturing with process steps in a different order is required: in addition to leukapheresis and activation of patient-specific T cells for viral transduction, unmodified T cells must first be expanded to achieve the appropriate cell number for experimental testing or clinical treatment and then transfected with CAR-encoding mRNA, followed by cryopreservation and reinfusion into the patient^3,11^. For time- and cost-effective production, automated and scalable processes are the central goal. Since all methods of ex vivo genetic modification of T cells involve some degree of cytotoxicity that can result in significant cell loss during manufacturing^12^, the activation and expansion of T cells are the critical steps to ensure a sufficient number of viable cells before and after mRNA transfer. Therefore, the optimal medium for mRNA-based CAR-T cells supporting cell viability, expansion, modification, and functionality as well as regulatory compliance for manufacturing under current Good Manufacturing Practice (cGMP) is one of the key parameters. Different protocols have evolved in the last decade^12–17^, since successful immunotherapy highly relies on selecting an appropriate culture medium to enable efficient expansion of the desired T cell type^18^. However, only a few protocols for clinical grade, GMP-compliant mRNA-based CAR-T cell manufacturing are available^11,19^. Here, in this study, we compared two different media protocols for CAR-mRNA-modified T cell generation using our current lab-scale process and evaluated their potential for upscaling in a closed system for clinical grade mRNA-based CAR-T cell therapies. Using ImmunoCult™-XF T Cell Expansion Medium (StemCell Technologies, Vancouver, Canada) and the recently released TheraPEAK® T-VIVO® medium (Lonza, Walkersville, USA) plus supplements recommended by each supplier, we compared expansion, viability, CAR-modification, T cell subsets, and functionality using primary T cells of four different healthy donors.

## Results

### Lab-scale workflow for mRNA-CAR-T cell generation

**Figure 1:**
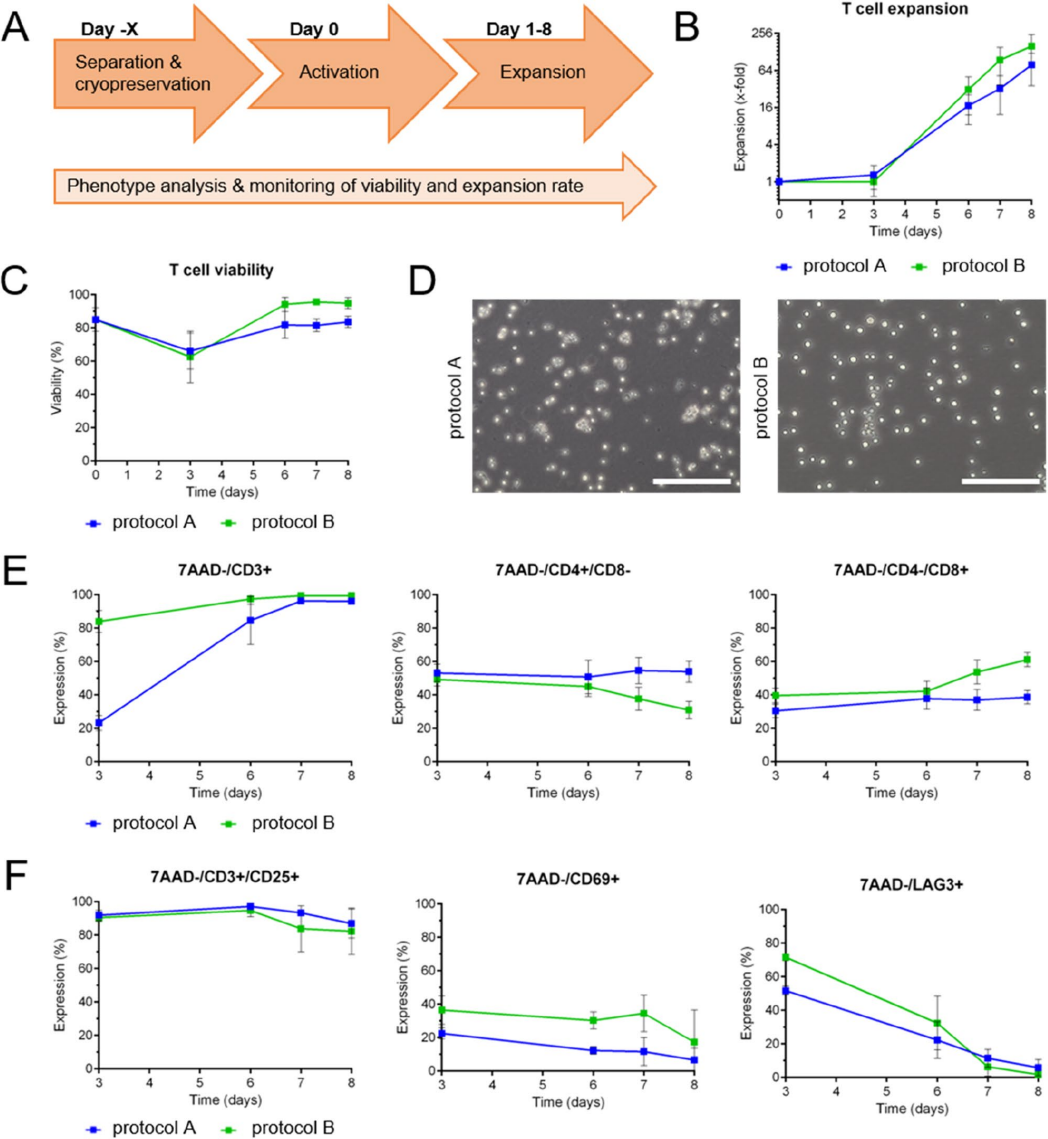
Experimental workflow, expansion rate, viability, and phenotyping of primary T cells during an 8-day culture process comparing two different media protocols. **(A)** Lab-scale process of mRNA-based CAR-T cell generation using primary T cells spanning separation (day -X), activation (day 0), and expansion (day 1–8). **(B,C)** Comparison of T cell expansion and viability during the 8-day culture process using protocols A (blue) and B (green). N = 4 independent experiments were performed. Values are displayed as the mean ± SEM. **(D)** Microscopic analysis of T cell morphology on day 7 after activation. One representative image each is displayed showing larger, more heterogeneous cell shapes in protocol A (blue) and a homogeneous, round-shaped cell population by using protocol B (green). Scale bar shows 200 μm. **(E,F)** Flow cytometric analysis of T cells during the 8-day culture process using protocols A and B. The percentage of marker expression is plotted against time in days. Analysis of T cell subsets **(E)** and activation status **(F)** revealed differences for both protocols tested. N = 4 independent experiments were performed. Values are displayed as the mean ± SEM.

**Figure 2:**
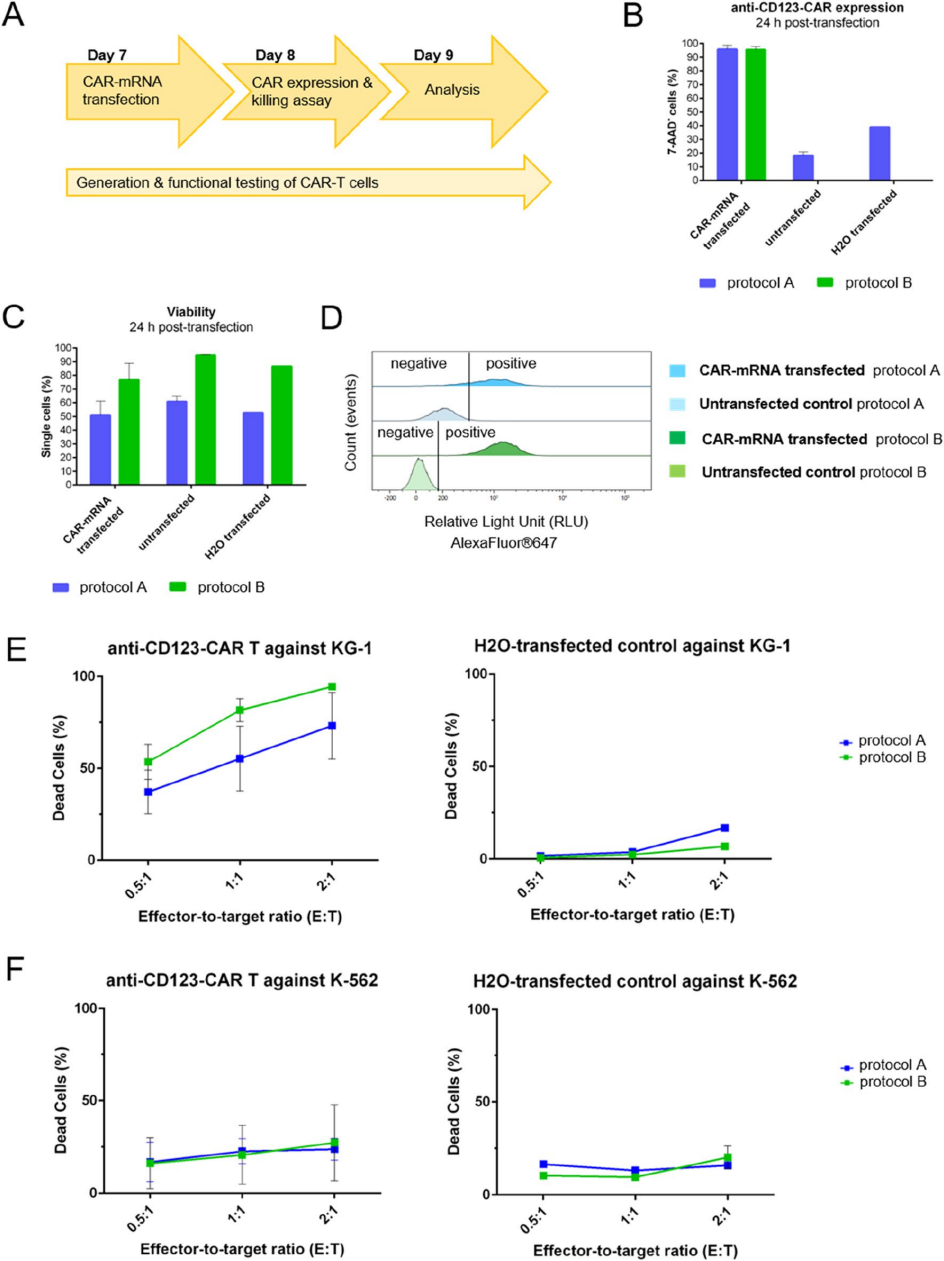
Comparison of anti-CD123 CAR expression, CAR-T cell viability, and CAR-specific killing activity 24 h post-transfection. **(A)** Experimental workflow for modification of expanded T cells with anti-CD123-CAR-mRNA via electroporation (day 7), read-out of CAR expression 24 h postelectroporation (day 8) and functional testing of CAR-T cells with read-out on day 9. **(B)** Twenty-four h post-transfection, CAR expression of T cells cultured in medium of protocol A (blue) or protocol B (green) was determined via F(ab′)2 staining and flow cytometric analysis; n = 3 independent experiments of anti-CD123 CAR transfection were carried out. Untransfected cells (n = 2) and water-transfected cells (n = 1) were used as negative controls. Values are displayed as the mean ± SEM or mean ± SEM in the case of n = 1. **(C)** Viability of aCD123-CAR-T cells after transient transfection was determined by 7-AAD staining and flow cytometric analysis. Transfected as well as untransfected cells expanded with protocol A showed lower viability than (CAR)-T cells expanded with protocol B 24 h post-transfection. **(D)** Histograms of F(ab′)2 staining are clearly separated into F(ab′)2-positive and F(ab′)2-negative when CAR-T cells were cultured according to protocol B (light and dark green), while histograms of CAR-positive and CAR-negative T cells overlap when grown by using protocol A (light and dark blue). **(E/F)** The percentage of dead target cells eliminated by aCD123-CAR-T cells or water-transfected control T cells is blotted against E:T ratios of 0.5:1, 1:1, and 2:1. T cells cultured according to protocol A are shown as blue curves, and those cultured according to protocol B are shown as green curves. For aCD123-CAR-T effector cells, the results of n = 4 independent experiments carried out in technical triplicates are depicted. For process control, water-electroporated T cells with n = 1 as a control experiment in technical triplicates are shown. All values are displayed as the mean ± SEM or mean ± SEM in the case of n = 1.

In general, mRNA-based CAR-T cell generation in a lab-scale procedure requires four main steps: isolation of primary T cells from healthy donors, T cell activation, expansion, and mRNA transfection. To investigate the optimal timepoint of mRNA uptake for CAR-T cell generation, T cells were expanded using protocol A and analyzed for cell numbers, expansion rates, viability, T cell subsets, transfection efficiency, and CAR expression levels. Considering an appropriate cell number for experimental testing (and later clinical treatment), high CAR-T cell viability, and high efficacy due to activation status, we defined day 6–10 as most suitable for mRNA transfection of stimulated T cells to generate transient CAR-T cells (summarized in Supplemental Fig. S1), which led to the experimental workflow that is presented in Figs. 1A and 2A.

### T cell expansion and viability

Primary T cells were isolated from PBMCs of four different healthy donors, cryopreserved, and T cell purity was analyzed using flow cytometry (day -X). Although lymphocyte composition varies across human individuals, the cell type frequency and T cell purity were in the anticipated range for all four donors used in this study^20–22^ (Supplemental Fig. S2). On day 0, T cells were thawed, monitored over an 8-day expansion process and compared regarding expansion rates, viability, phenotype, mRNA-based CAR-modification, and functionality using two different media protocols.

The initial parameter examined in this study was the expansion rate of T cells after thawing and activation. Although the T cells from each donor showed individual growth behavior and adaptation to the respective conditions (Supplemental Fig. S3), across all donors, protocol B tended to provide a higher T cell number on day 8 (158.3x-fold ± 75.3) than protocol A (78.7x-fold ± 37.1) (Fig. 1B). Trypan blue staining of the cells using both protocols revealed decreased viability on day 3: protocol A, day 0 85.0% ± 6.1 vs. day 3 66.1% ± 9.4; protocol B, day 0 85.0% ± 6.1 vs. day 3 62.6% ± 13.4. On day 6, the cells were recovered, as indicated by the same or even higher viability than after isolation (> 80% viability). A direct comparison of protocols A and B depicted a 10–20% higher viability for protocol B on day 6 (protocol A 81.8% ± 7.0; protocol B 94.2% ± 3.7) (Fig. 1C). This is underlined by the microscopic analysis showing differences in cell morphology on day 7. While cells cultured according to protocol B showed round shaped cells, T cells cultured with protocol A appeared larger, slightly deformed and showed more cell debris (Fig. 1D).

### Characterization of T cells after activation

To investigate how different protocols influence the composition of the T cell populations and the activation status, T cell subsets and selected activation/exhaustion markers were monitored from day 3 to day 8 by flow cytometry. The most obvious difference was obtained for the CD3 status of the T cells at day 3 (protocol A 23.3% ± 3.9; protocol B 84.0% ± 5.7), mainly due to the different activation reagents used in the two protocols (Fig. 1E, left panel). The recommended activator reagent used in protocol A consists of a mixture of soluble T cell activating antibodies (anti-CD2/anti-CD3/anti-CD28) binding the surface receptors on T cells, leading to internalization of CD3^23^. In contrast, protocol B uses anti-CD3/anti-CD28 antibodies immobilized on a polymeric nanomatrix, leading to T cell activation but no internalization of CD3 surface receptors into T cells. Therefore, a higher CD3 signal was obtained for protocol B directly after T cell activation (days 3–6). However, CD3 levels aligned to > 90% CD3-positive cells for both protocols at day 7 (protocol A 96.3% ± 0.4; protocol B 99.5% ± 0.0) (Fig. 1E, left panel). For the distribution of T helper and cytotoxic T cells among the CD3-positive fraction, CD4- and CD8-expression levels were analyzed for both media protocols. While protocol A showed higher levels of CD4^+^/CD8^−^ T helper cells at day 7 (protocol A 54.6% ± 6.8; protocol B 37.7% ± 6.0), protocol B promoted the formation of higher levels of CD4^−^/CD8^+^ cytotoxic T cells (protocol A 37.0% ± 5.4; protocol B 53.7% ± 6.2) (Fig. 1E, middle, right panel).

For evaluation of the T cell activation status, CD25 expression was analyzed, which resulted in comparable and stable surface expression levels for both protocols with a slight decrease from day 6 after activation to ≥ 80% (Fig. 1F, left panel). CD69 expression, an indicator of early T cell activation, decreased on T cells within both media from day 3 to day 8 after activation, indicating a decline in activation status (Fig. 1F, middle panel). Lymphocyte-activation gene 3 (LAG-3) was used as a representative marker for T cell exhaustion. The portion of LAG3^+^ cells decreased during expansion from day 3 to day 8 after activation, indicating a higher exhaustion of the T cells directly after activation with T cell activator reagents (Fig. 1F, right panel).

### T cell transfection and CAR expression determination

To investigate the optimal protocol for mRNA delivery to T cells via electroporation in general, the electroporation workflow was established using protocol A. Therefore, different electroporation settings recommended by the manufacturer were compared for the highest transgene expression while preserving the best T cell viability. In initial experiments, T cell electroporation was carried out using a reporter mRNA encoding enhanced green fluorescent protein (EGFP), and the top three settings showing the highest EGFP expression and cell viability were determined (Supplemental Fig. S4). The findings were verified with an mRNA encoding anti-CD123-CAR and GFP (constructs shown in Supplemental Fig. S5), and pulse code CM-138 was revealed as the most suitable setting to transiently transfect T cells with high transgene expression and preserved viability (Supplemental Fig. S6). This pulse code was then defined as the standard electroporation setting for anti-CD123-CAR-mRNA transfection in the present protocol comparison.

To assess the number of CAR-positive T cells after transient transfection using CAR-mRNA electroporation, CAR surface expression was determined on day 8 (24 h post-transfection) via F(ab′)_2_-goat anti-mouse IgG antibody staining—an antibody recognizing the antigen binding part (scFv) of the CAR protein serving as a universal CAR detection reagent^24^. The CAR expression levels were similar for both T cell expansion protocols at 24 h post-transfection. At 48 h post-transfection, the CAR expression always dropped by at least 50% independent of the protocol used for expansion and transfection, even with the optimal pulse code used for electroporation (Supplemental Fig. S6) and at 72 h post-transfection no CAR expression was detected anymore (data not shown). However, other technologies such as lipid nanoparticle delivery of CAR-mRNA are currently under investigation, indicating a prolonged CAR expression and CAR-T cell functionality until day 6 post-transfection (EP vs. LNP data, manuscript accepted^25^). For untransfected T cells, as well as water-transfected T cells as electroporation process controls, unspecific signals were observed on cells cultivated according to protocol A but not for T cells expanded with protocol B. This indicates unspecific binding of the F(ab′)_2_ antibody used for CAR detection to other F(ab′)_2_ structures only present in protocol A (Fig. 2B). While protocol A uses a T cell activating reagent comprising soluble anti-CD2/CD3/CD28 antibodies, protocol B uses nanomatrix bound anti-CD3/CD28 antibodies for T cell activation, which can be more easily removed than soluble antibodies remaining on the cell surface interfering with CAR staining using the F(ab′)_2_-goat anti-mouse IgG antibody. The histograms of CAR-mRNA-transfected T cells (AlexaFluor®647-positive) and untransfected T cells (AlexaFluor®647 negative) expanded according to protocol A slightly overlap, while a clear separation of CAR-expressing T cells (AlexaFluor®647 positive) and non-CAR-expressing T cells (AlexaFluor®647-negative) expanded with protocol B is ensured (Fig. 2D).

The effect of transfection with anti-CD123-CAR-mRNA on T cell viability was assessed on day 8 24 h post-transfection. Comparing CAR-mRNA-transfected and untransfected T cells of the same expansion protocol, the untransfected control T cells showed a 15–20% higher viability than the CAR-mRNA transfected T cells: protocol A (51.0% ± 8.9), protocol B (76.8% ± 10.3) (Fig. 2C). The viability of water-transfected T cells is slightly decreased in comparison to untransfected T cells: protocol A (60.8% ± 2.9), protocol B (94.7% ± 0.2), indicating that the electroporation process itself only slightly affects T cell viability, whereas the introduction of mRNA into the cells has the more drastic effect on viability irrespective of the protocol used for expansion (Fig. 2C). Comparing the two protocols, CAR-mRNA- and water-transfected as well as untransfected T cells cultured with protocol A showed lower viability than T cells cultured with protocol B (Fig. 2C), which aligns with the viability data obtained on days 6 and 7 of untransfected T cells (Fig. 1C). Since the percentage of viable cells after CAR-mRNA electroporation is much higher (76.8% ± 10.3 vs. 51.0% ± 8.9), protocol B is more suitable for clinical use than protocol A. Currently, one FDA-approved dose is 0.1 to 6 × 10^8^ CD19-directed CAR-positive viable T cells per kg of body weight for patients > 50 kg, depending on the indication to be treated^26^. If this is applied to transient CAR-T cells, it requires the production of approximately 9 × 10^8^ CAR-T cells for one dose of viable CAR-T cells at 76.8% viability (Fig. 2C), which is feasible, even if more doses for multiple applications are required.

### Functionality of transient anti-CD123-CAR-T cells

After modification of primary T cells with an anti-CD123-CAR, the transient CAR-T cells were analyzed for their cytotoxicity against tumor cells. Therefore, 24 h co-cultures with the CD123-positive AML cell line KG-1 or the CD123-negative cell line K-562 as a control were set up. After 24 h of co-culture, dead target cells were determined via staining with 7-AAD. Anti-CD123-CAR-T effector cells cultured according to protocol A led to less KG-1 cell death at all E:T ratios tested compared to protocol B (Fig. 2E). T cells electroporated with water served as process control and showed no or minimal target cell death (Fig. 2E). As an off-target control, anti-CD123-CAR-T cells were co-cultured with CD123-negative chronic myelogenous leukemia K-562 cells, and no CAR-specific cytotoxicity was observed when compared with the water-transfected T cells (Fig. 2F).

## Discussion

To overcome safety concerns and time- and cost-intensive manufacturing processes under higher biosafety levels, mRNA-based, transient CAR-T cell generation is an attractive alternative approach to the currently approved CAR-T products, which are generated by viral transduction^27–30^.

The critical step in the CAR-T cell generation procedure is T cell activation and expansion to ensure sufficient numbers of viable cells for experimental testing or clinical treatment and effective CAR-T functionality after modification. Due to the transient character of CAR expression after mRNA transfection, CAR-T cell “wellbeing” is even more critical than for virally transduced CAR-T cells that maintain CAR expression after each cell division cycle.

Transient CAR-T cells have been shown to be effective in several preclinical studies and have shown preliminary evidence of anti-tumor efficacy in clinical trials^31^. However, there is still a need to optimize the manufacturing process, since mRNA-based CAR-T cells require the application of multiple doses for an effective anti-tumor response^32^. This in turn requires the production and storage of several batches, which can only be achieved by optimizing expansion conditions to generate the highest number of the most viable and effective CAR-T cells possible.

Although a few protocols have been published comparing different media and reporting the generation of functional CAR-T cells in vitro^12–17^*,* a time- and cost-effective production process already considering the later use of automated and scalable GMP-compliant manufacturing of mRNA-based CAR-T cells is lacking. Therefore, we thought one step ahead and designed a lab-scale process for mRNA-based CAR-T cell generation using electroporation as an example process for transient transfection, compared two media protocols, and evaluated their potential for upscaling and process development of mRNA-based CAR-T cell therapies.

While both protocols enabled the production of viable and functional CAR-T cells in vitro, as reported for other media tested in several previous studies^12–17^, our lab-scale study revealed differences in expansion rate, viability, T cell subsets, CAR detection accuracy, and CAR-T cell efficacy. We determined that protocol B is best suited to generate optimal mRNA-CAR-T cells and defined the following criteria that must be met to obtain a high-quality mRNA-CAR-T cell product:

1. High T cell numbers, high viability, and activation status prior to transfection. Large numbers of highly viable and activated T cells are especially important since current manufacturing processes use patient-derived T cells from mostly heavily pre-treated patients with poor T cell quality. For the mRNA approach, an expansion step prior to mRNA modification is included. This expansion step of T cells for 6–10 days is in advantage of high T cell numbers, viability, and proper activation status which, in turn, leads to high-quality transient CAR-T cells. Since patients are heavily pre-treated when they receive approved CAR T cell products as 2nd or 3rd line therapy, the quality of the starting material is often very poor with low viability and lymphocyte collection efficiencies^33,34^. Furthermore, a T cell selection and enrichment prior genetic modification lowers the risk for modification of undesired cells, and contaminating red blood cells (RBCs), monocytes, and neutrophils in the starting material may adversely affect T cell expansion in culture as well as final CAR-T cell product characteristics^33^.
2. The mRNA should be delivered between day 6 and 10 after T cell activation, otherwise either cell numbers are not high enough, yet, or the T cells are no longer activated enough for being effective for a couple of days. Here, we defined day 7 to be the most suitable day for CAR-mRNA delivery.
3. The highest possible CAR-T cell functionality is required for efficient target cell killing. This can be supported by a high CD8:CD4 ratio, which promotes fast and efficient cytotoxicity. For virally transduced CAR-T cells, CD4^+^ CAR-T cells are described to be initially slower in tumor killing than CD8^+^ CAR-T cells, but are therefore less prone to exhaustion and therefore more persistent in the body after antigen exposure. The opposite is true for CD8+ CAR T cells^35^. However, these facts do not play a role in transient CAR-T cells. The transient nature of mRNA-CAR-T cells requires rapid and effective tumor killing and therefore high levels of CD8-positive cells, as long-term persistence of CAR-T cells does not need to be considered due to the short half-life of mRNA and transient CAR expression.

Only by using protocol B we achieved all the criteria for a high-quality transient CAR-T cell product in the lab-scale process, indicating that this protocol is best suited for upscaling. Using this optimal T cell expansion protocol B, we could show that PMBCs could be used as starting material for T cell enrichment (Supplemental Fig. S3B,C) without prior T cell separation via magnetic beads, as performed in all previous experiments (Figs. 1, 2). The T cell expansion rate and viability were comparable to isolated T cells, indicating that PBMCs might be a more time and cost-effective starting material saving additional purification steps and costly consumables for T cell isolation prior expansion. Furthermore, this procedure has been shown to be effective for virally transduced CAR-T generation using automated systems^36–38^, suggesting that a similar procedure may be conceivable for mRNA CAR-T cells.

In a next step, we will transfer this transient CAR-T protocol to an upscaling procedure and process development into a GMP-ready process using automated systems. As shown for virally transduced CAR-T cells, automated systems such as the CliniMACS Prodigy device are the next-generation methods to produce CAR-T cells in a time- and cost-effective manner^38,39^. Even gene-edited CAR-T cells delivering Cas9-mRNA and guide RNAs^40^ or the transposon-based sleeping beauty technology^41^ via electroporation were successfully manufactured in the CliniMACS Prodigy, indicating the high relevance of automated systems towards clinical translation.

We are aware that lab-scale protocols such as our procedure described here are often intended for translation into GMP, but the actual performance under GMP- or GMP-like conditions mostly requires further optimization or even adaptation^11^. However, even for lab-scale we already considered GMP-upscaling-compliancy from the beginning on. Although the transfection method used for this study relies on electroporation, we have this optimal lab-scale mRNA-CAR-T cell process in hand as well as a lipid nanoparticle (LNP)-based transfection method (comparison of electroporation vs. LNP-based transfection, manuscript accepted^25^), and we are best equipped with automation devices and experienced team for the next level to ultimately determine the most suitable protocol for mRNA-CAR-T cell manufacturing for clinical trials.

## Methods

### Experimental design

#### Primary T cell isolation, expansion, and cell culture

Primary T cells were isolated from human peripheral blood mononuclear cells (PBMCs) of healthy donors collected by the Institute for Transfusion Medicine of the University Clinic of Leipzig, Germany, approved by the Ethics Committee of the Faculty of Medicine of the University Clinic of Leipzig, Germany, as no ethical or scientific concerns were raised (file reference number: 272-12-13082012). Informed consent was obtained from all healthy donors and/or their legal guardian(s). Furthermore, we confirm that all experiments were performed in accordance with relevant guidelines and regulations.

PBMCs were extracted from donor material using Ficoll gradient centrifugation, and immune phenotyping was performed by flow cytometry. T cell isolation was achieved by magnetic separation using the Pan T Cell Isolation Kit (Miltenyi Biotech, Bergisch Gladbach, Germany). Cell viability and quality of separation were assessed by trypan blue staining and flow cytometry, respectively. Subsequently, 5 × 10^6^ T cells were frozen and stored in liquid nitrogen until day -X. For protocol comparison, frozen T cells were thawed (day 0), taken up in the media of the respective protocol, activated and expanded for 8 days.

For expansion, cells were either cultivated in ImmunoCult™-XF T Cell Expansion Medium or in TheraPEAK® T-VIVO® medium, IL-2 supplemented and activated according to suppliers’ specifications. In detail, ImmunoCult™-XF T Cell Expansion Medium was supplemented with 218 IU/mL IL-2 (PeproTech, London, UK), and T cells were activated by 25 µL/mL ImmunoCult™ Human CD3/CD28/CD2 T Cell Activator reagent at a cell density of 1 × 10^6^ cells/mL (StemCell Technologies, Vancouver, Canada) (referred to as protocol A). TheraPEAK® T-VIVO® was supplemented with 100 IU/mL IL-2 (Novartis, Basel, Switzerland), and T cells were activated by 10 µL/mL T Cell TransAct™ reagent at a cell density of 1 × 10^6^ cells/mL (Miltenyi Biotech, Bergisch Gladbach, Germany) (referred to as protocol B).

For both protocols, T cells were activated on day 0 and incubated at 37 °C and 5% CO_2_ for up to 8 days. From days 3 to 8, T cells were monitored regarding cell number using cell counting chambers, cell viability by trypan blue staining, and T cell subsets by flow cytometry. The cell density was adjusted to 1.0–2.5 × 10^5^ cells/mL by adding fresh medium.

KG-1 and K-562 cells (both DSMZ, Braunschweig, Germany) were cultured in RPMI1640 media supplemented with 10% heat-inactivated fetal calf serum (*v/v*).

#### Flow cytometry for T cell characterization

To assess the quality of T cell isolation, cells were fluorescently stained with anti-CD45-V500, anti-CD3-FITC, anti-CD4-APC-H7, anti-CD8a-PE, anti-CD19-APC, anti-CD14-PerCP, and anti-CD56-PE-Cy7 antibodies (Beckton Dickinson, Franklin Lakes, USA). For monitoring T cell subsets (day 3 to day 8), cells were fluorescently stained with anti-CD3-FITC, anti-CD4-BV510, anti-CD8-PE-Cy7, anti-CD127-AlexaFluor®647, anti-CD25-PE, anti-CD69-APC-H7, and anti-LAG-3-BV421 antibodies (Beckton Dickinson, Franklin Lakes, USA). Briefly, cells were harvested, washed, resuspended in 1× phosphate buffered saline (PBS) at pH 7.4, and incubated with antibodies for 30 min at 4 °C. After antibody incubation, cells were washed with PBS, incubated with 7-aminoactinomycin (7-AAD) reagent 10 min prior to measurement and analyzed via flow cytometry as described below.

All cells were analyzed by flow cytometry using BD FACSCanto™ II (Beckton Dickinson, Franklin Lakes, USA). Dead cells and cell debris were excluded from the analysis based on 7-AAD fluorescence and scatter signals. Analysis was performed using Kaluza flow cytometry analysis software (Beckman Coulter, Brea, USA).

#### mRNA transcripts

A reporter mRNA encoding enhanced green fluorescent protein (EGFP), including 5-methoxyuridine (5-moU) base modification, CleanCap™ cap 1 structure, and poly(A) tail was purchased as fully processed mRNA from TriLink, San Diego, USA. The CAR-mRNA was synthesized via in vitro transcription (IVT) using a T7 promotor-containing DNA construct produced by PCR from plasmid DNA encoding a second generation anti-CD123-CAR ± GFP (Creative Biolabs, New York, USA) as template. A schematic of the mRNA constructs is shown in Supplemental Fig. S5. According to the manufacturer’s instructions, 1 µg of template DNA was transcribed into RNA by a HighYield T7 RNA synthesis kit (Jena Biosciences, Jena, Germany) using T7 RNA polymerase at 37 °C for 4 h. For mRNA modification, 5 mM uridine 5ʹ-triphosphate (UTP) was replaced by an equivalent amount of 1-*N*-methyl pseudouridine (m1Ψ) 5ʹ-triphosphate (Jena Biosciences, Jena, Germany). Capping of the IVT mRNA was performed cotranscriptionally by using 5 mM CleanCap® Reagent AG (3ʹ OMe) (TriLink, San Diego, USA). Subsequently, DNA template removal was achieved by adding 2 U DNase for 15 min at 37 °C. After purification, 10 µg mRNA was processed by 250 U/mL *E. coli* poly(A) polymerase and 1 mM adenosine 5ʹ-triphosphate (ATP) supplemented with RNase-inhibitor for 60 min at 37 °C. The m1Ψ-modified mRNA was isolated from the reaction mix by a NucleoSpin RNA Clean-up Kit (Macherey–Nagel, Düren, Germany) and eluted in nuclease-free water as recommended by the manufacturer. The RNA concentration was determined using a Nanodrop 2000c spectrophotometer (Thermo Fisher Scientific, Waltham, USA). Capped and poly(A)-tailed mRNAs were analyzed by agarose gel electrophoresis containing 0.3% hydrogen peroxide (*v/v*).

#### Transient transfection and CAR expression analysis

On day 7, expanded T cells were transiently transfected with anti-CD123-CAR ± GFP mRNA using the 4D-Nucleofector™ X Unit and P3 Primary Cell 4D X Kit L (Lonza, Walkersville, USA). For optimization of electroporation parameters, commercially available EGFP-mRNA and anti-CD123-CAR + GFP were used, while for media testing, anti-CD123-CAR without GFP was used. A total of 3 × 10^6^ T cells were resuspended in 90 µL 4D-Nucleofector™ Solution, combined with 6 µg of mRNA, transferred to Nucleocuvette™ vessels, electroporated at a defined pulse code (see Supplemental Figs. S4, S6), resuspended in 500 µL from the respective media and cultured in a 24-well plate at 37 °C and 5% CO_2_.

Twenty-four hours post-transfection, CAR expression levels were measured by an AlexaFluor®647-conjugated goat F(abʹ)_2_ antibody directed against mouse IgG-F(abʹ)_2_ fragments (Jackson ImmunoResearch Laboratories, West Grove, USA). This staining allows the cell surface detection of the scFv within the CAR independently of its target antigen, thus serving as a universal CAR detection method^24^. Therefore, cells were collected, washed, resuspended in 1× PBS, incubated with F(ab′)_2_-goat anti-mouse IgG antibody for 60 min at 4 °C, washed with 1× PBS, and analyzed using flow cytometry.

#### CAR-T functionality test (killing assay) at day 8 (24 h post-transfection)

CD123^+^ (KG-1) and CD123^−^ (K-562) target cells were stained using the CellTrace™ Violet Cell Proliferation Kit (Thermo Fisher Scientific, Waltham, USA) according to the manufacturer’s specifications and co-cultured with anti-CD123-CAR-T effector cells at different effector:target (E:T) ratios (2:1, 1:1, 0.5:1) at 37 °C and 5% CO_2_ for 24 h. Target cells only cultivated in RPMI 1640 medium supplemented with 10% heat-inactivated fetal calf serum (*v/v*) served as negative control, while target cells treated with 0.2% Tween 20 solution in medium (*v/v*) were used as positive control. After 24 h of co-culture, cells were harvested and incubated with 7-AAD. Target cell killing was determined by monitoring dead cells via 7-AAD in the CellTrace^TM^ Violet-stained target cell population by flow cytometry.

### Supplementary Information

Supplementary Figures.

## Data Availability

The authors declare that all data in support of the main findings of this study are available within the paper and its Supplementary Information files.
