# Supplementary material for: Comparison of two lab-scale protocols for enhanced mRNA-based CAR-T cell generation and functionality
Source: Sci Rep. 2023 Oct 24;13:18160. doi: 10.1038/s41598-023-45197-x (PMC10598065; doi:10.1038/s41598-023-45197-x)
Supplement: Supplementary file 1 — Supplementary Figures. [file 41598_2023_45197_MOESM1_ESM.pdf]

Comparison of two lab-scale protocols for enhanced mRNA-based CAR-T cell generation and functionality

Nadine von Auw[1], Robert Serfling[1], Reni Kitte[1], Nadja Hilger[1], Chengkang Zhang[2], Clara Gebhardt[1], Anna Duenkel[1], Paul Franz[1], Ulrike Koehl[3,4,5], Stephan Fricke[1,3], U. Sandy Tretbar[1,3*]

[1]Fraunhofer Institute for Cell Therapy and Immunology (IZI), Department for Cell and Gene Therapy Development, Perlickstr. 1, 04103 Leipzig, Germany

[2]Lonza, 9900 Medical Center Drive, Rockville, MD 20850, USA

[3]Fraunhofer Cluster of Excellence Immune-Mediated Diseases CIMD, Leipzig, Germany

[4]Fraunhofer Institute for Cell Therapy and Immunology (IZI), Institute's Director, Perlickstr. 1, 04103 Leipzig, Germany

[5]Institute for Clinical Immunology, Medical Faculty, University of Leipzig, Johannisallee 30, 04103 Leipzig, Germany

[*]corresponding author

Supplemental Information

Supplemental Figures:

Figure S1:

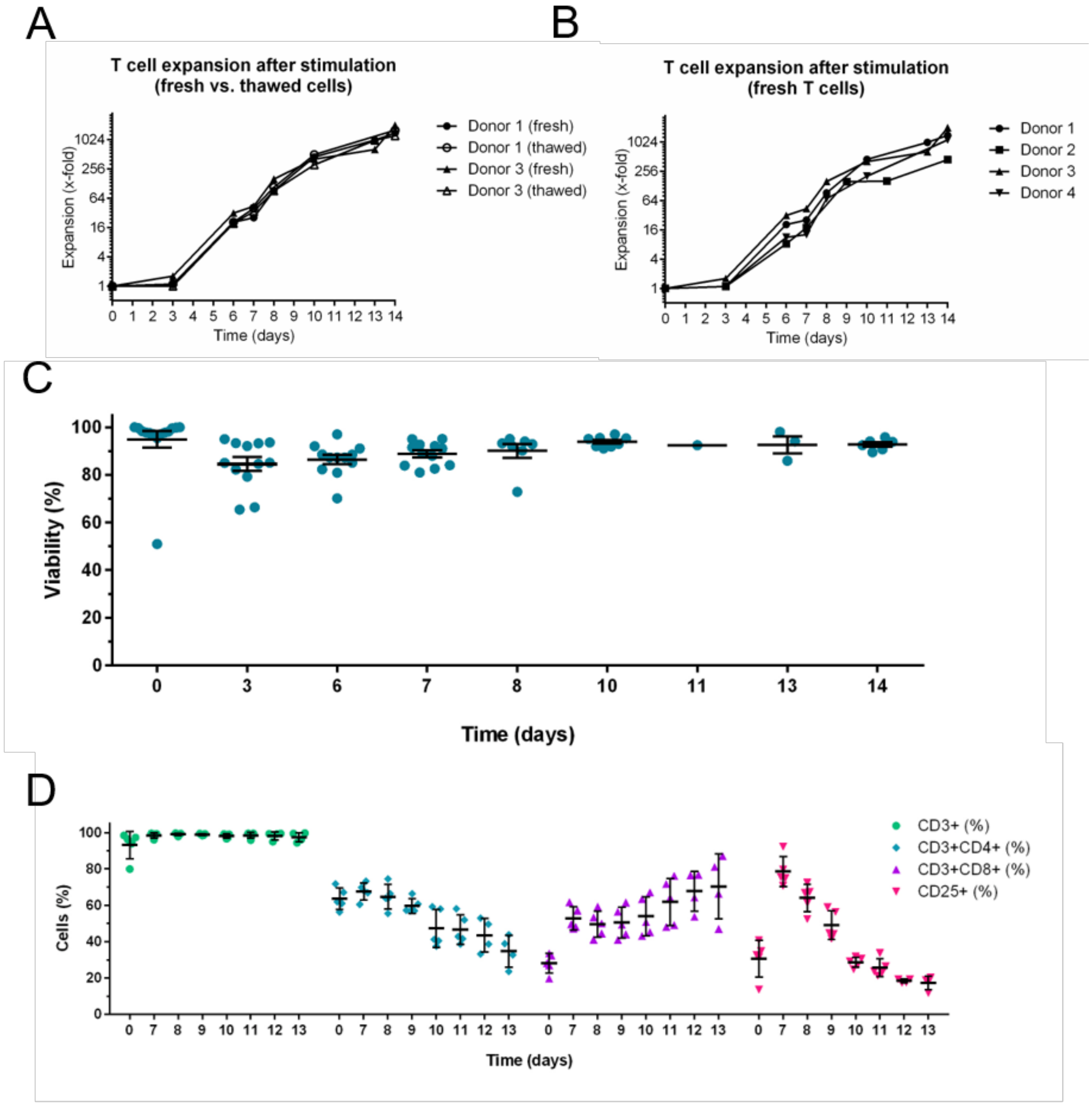

Figure S1. Monitoring of T cell expansion, viability, and activation status after isolation and stimulation. (A) The expansion of T cells from two healthy donors was compared when cultured directly after isolation or expanded after thawing from frozen culture. **(B)** Primary T cells of four healthy donors were cultured according to protocol A and monitored for cell expansion over 14 days. (**C**) 14 different T cell cultures were checked for viability and available data points were plotted. **(D)** T cell cultures were monitored for up to 13 days regarding T cell subsets and activation and available data points were plotted. The results of n = 5 independent experiments carried out in technical triplicates are depicted. All values are displayed as the mean ± SEM.

Figure S2:

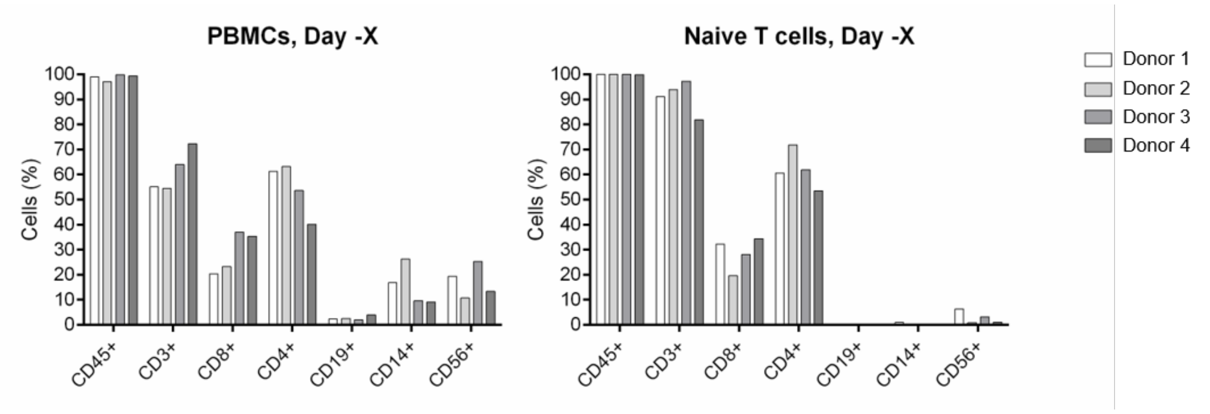

Figure S2: Quality control of T cell isolation. Since the frequency of lymphocytes varies across individuals, the quality of T cell separation was assessed on the day of isolation (day -X) by an immune cell phenotyping using flow cytometry. After Ficoll gradient, immune phenotyping of the obtained peripheral blood mononuclear cells (PBMCs, day -X) showed an adequate composition of 45-70 % T cells (CD3+), 5-10 % B cells (CD19+), 5-10 % monocytes (CD14+), and 5-30 % NK cells (CD56+). CD3+ T cells were composed of CD4+ and CD8+ cells, roughly in a 2:1 ratio. After T cell isolation, naïve T cells were analyzed using the same antibody panel for immune phenotyping, indicating high purity of the T cell population by CD3+/CD4+/CD8+ cells. References for cell numbers: (1–3).

S3:

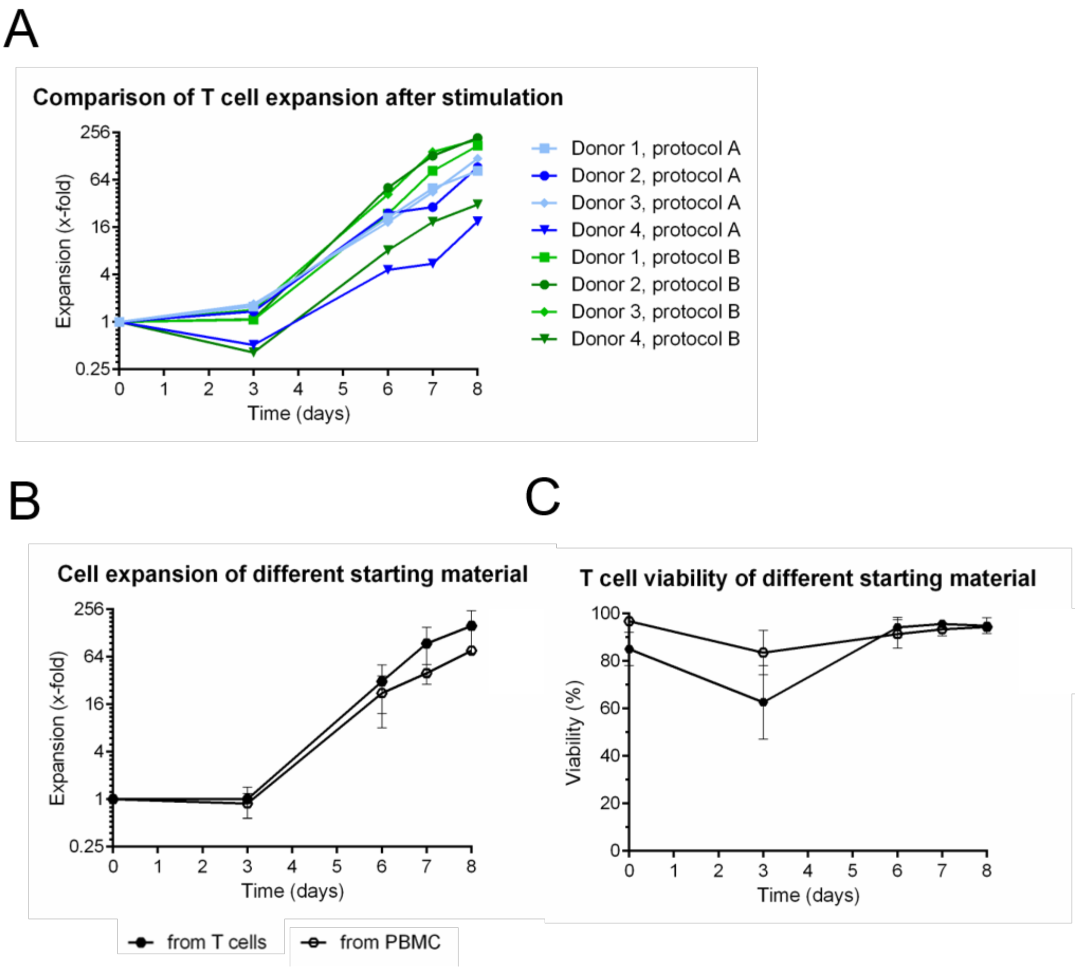

Figure S3: Comparison of Cell expansion rates and viability of different staring material. (A) Primary T cells of four healthy donors were monitored for cell expansion over eight days. Cells were either cultured at 37 °C and 5 % CO_2 according to protocol A (blue) or protocol B (green). Values are displayed as one biological experiment (n=1). (B, C) Using protocol B, cell expansion and viability of different starting material (isolated T cells or PBMC) were compared over eight days at 37 °C and 5 % CO_2.

Figure S4:

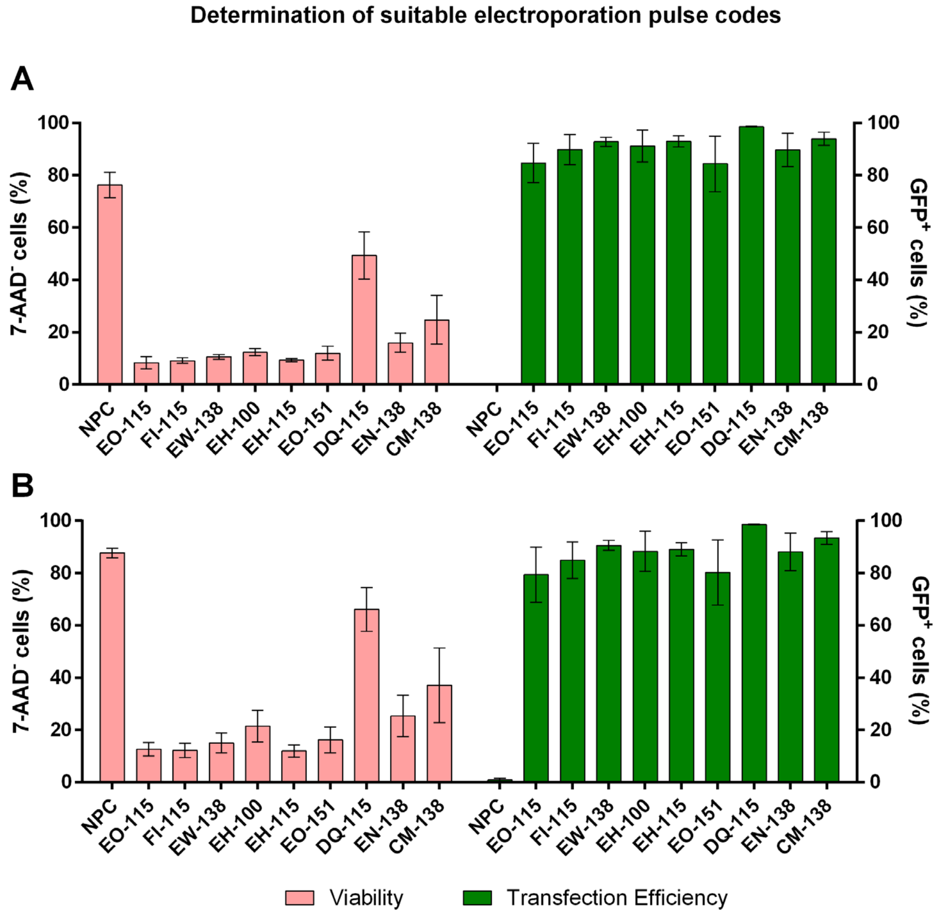

Figure S4: Electroporation of primary T cells with EGFP-mRNA using different electroporation parameters (pulse codes). Freshly prepared T cells were stimulated and cultured according to protocol A up to seven days before electroporation with commercially available EGFP-mRNA (TriLink). A total of nine different pulse codes were tested. The transfection efficiency (percentage of EGFP-positive cells (GFP^+)) and viability (percentage of 7-AAD-negative cells (7-AAD^-)) were measured via flow cytometry 24 h (A) and 48 h (B) after transfection. Untransfected cells mixed with mRNA were used as non-pulse control (NPC). Data represent the means (±SEM) of two replicates from one donor, each measured as technical duplicates.

Figure S5:

aCD123-CAR-mRNA:

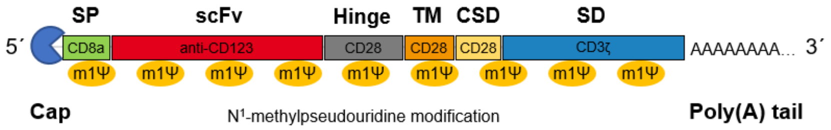

aCD123-CAR-P2A-GFP-mRNA:

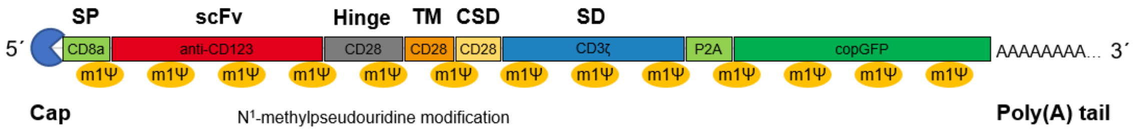

EGFP-mRNA:

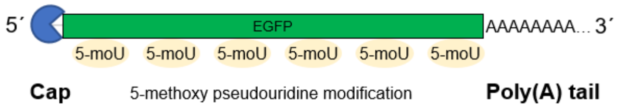

Figure S5: Schematic of the mRNA constructs used in this study. For transient transfection, an anti-CD123-CAR-mRNA and a reporter EGFP-mRNA were used. Both were capped, poly(A)-tailed, and modified with pseudouridine variants instead of uridine 5'-triphosphate (UTP). The CAR-encoding sequence consists of signal peptide (SP), CD123-directed single chain variable fragment (scFv), CD28 hinge, CD28 transmembrane domain (TM), CD28 co-stimulatory domain (CSD), and a CD3ζ signaling domain (SD). The reporter EGFP-mRNA was only used for pulse code testing for optimal electroporation setting determination.

Figure S6:

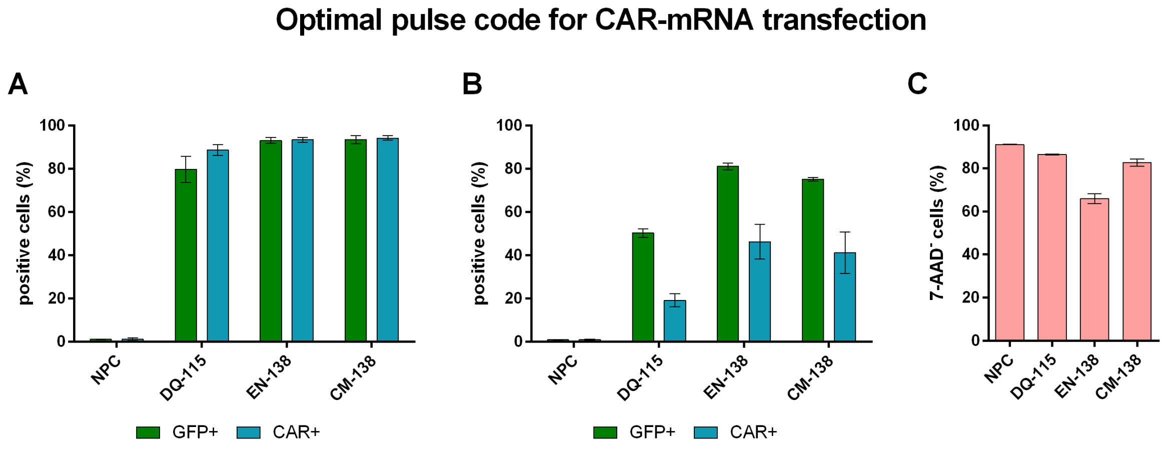

Figure S6: Electroporation of primary T cells with aCD123-CAR-P2A-GFP model mRNA using three pre-selected pulse codes. Freshly prepared T cells were stimulated and cultured for up to 10 days before electroporation with CAR-mRNA. Untransfected cells mixed with mRNA were used as non-pulse control (NPC). Data represent the means (±SEM) of two replicates from one donor, each measured as technical duplicates. The transfection efficiency (percentage of EGFP-positive cells (GFP^+)) and CAR expression was measured via flow cytometry 24 h **(A)** and 48 h **(B)** after transfection. **(C)** The viability was determined 24 h after transfection as a percentage of 7-AAD-negative ($7\text{-}AAD^-$) cells: NPC (no pulse control) 91.1% ± 0.1%; DQ-115 86.6% ± 0.0%; EN-138 66.1% ± 3.5%; 82.7% ± 2.2%. Data represent the means (±SEM) of two replicates from one donor, each measured as technical duplicates.

References

1. Daniel Lozano-Ojalvo Rosina López-Fandiño and Iván López-Expósito. "PBMC-Derived T Cells: Chapter 16,". In: Verhoeckx K, Cotter P, López-Expósito I, Kleiveland C, Lea T, Mackie A, et al., editors. *The Impact of Food Bioactives on Health: in vitro and ex vivo models: Peripheral Blood Mononuclear Cells*. Cham (CH) (2015).
2. Miltenyi Biotec. *Blood: Subtypes of PBMCs*. Available from: https://www.miltenyibiotec.com/DE-en/resources/macs-handbook/human-cells-and-organs/human-cell-sources/blood-human.html#gref
3. Kleiveland CR. *The Impact of Food Bioactives on Health: in vitro and ex vivo models: Peripheral Blood Mononuclear Cells*. Cham (CH) (2015).
